# Anaesthetic Management and Physiologic Effects of Pneumoperitoneum in Patients With Chronic Obstructive Pulmonary Disease Undergoing Laparoscopic Cholecystectomy

**DOI:** 10.7759/cureus.46458

**Published:** 2023-10-04

**Authors:** Neeraj Gautam, Mamta Harjai, Parul Sharma, Sujeet Rai, Manoj Tripathi, Deepak Malviya, Arvind Kumar Singh

**Affiliations:** 1 Critical Care, Sir Ganga Ram Hospital, New Delhi, IND; 2 Anesthesiology and Critical Care, Dr. Ram Manohar Lohia Institute of Medical Sciences, Lucknow, IND; 3 Community Medicine, Dr. Ram Manohar Lohia Institute of Medical Sciences, Lucknow, IND

**Keywords:** end-tidal co2, physiological changes, pneumoperitoneum, laparoscopic cholecystectomy, chronic obstructive pulmonary disease

## Abstract

Objective: This study aimed to assess the physiological changes and clinical outcomes in patients with chronic obstructive pulmonary disease (COPD) undergoing laparoscopic cholecystectomy.

Methods: This prospective cohort study included 50 patients of the American Society of Anesthesiology (ASA) physical status I and II with mild to moderate COPD (Global Initiative for Chronic Obstructive Lung Disease (GOLD) stage I-II) scheduled for laparoscopic cholecystectomy. We monitored heart rate, mean arterial pressure, end-tidal carbon dioxide (EtCO_2_), arterial carbon dioxide (PaCO_2_), and bicarbonate (HCO_3_) levels at baseline, 30 minutes after induction or 15 minutes post-insufflation, 15 minutes post-deflation, and 60 minutes post-operative. Perioperative complications and post-operative recovery characteristics were also observed. Descriptive statistics were used to summarise the demographic and clinical characteristics of the patients. The correlation between HCO_3_ and EtCO_2 _was plotted on a scatterplot, and Pearson’s correlation ‘r’ was calculated. The changes in physiological parameters over time were analysed using a paired t-test. A p-value of less than 0.05 is considered statistically significant.

Results: We observed a statistically significant but transient increase in heart rate, mean arterial pressure, and EtCO_2 _at 30 minutes after induction or 15 minutes post-insufflation, which returned to baseline levels within 15 minutes of deflation. Similarly, arterial CO_2_ and bicarbonate levels were also significantly increased at 15 minutes post-insufflation, yet remained within the normal physiological range. The study reported no serious perioperative complications, and all patients had an uneventful recovery.

Conclusion: While patients with mild to moderate COPD can experience transient physiological changes during laparoscopic cholecystectomy, these changes are generally well-tolerated and not associated with adverse clinical outcomes. Therefore, laparoscopic cholecystectomy can be considered a safe procedure in these patients. Future research should focus on the implications and safety of this procedure in patients with severe COPD.

## Introduction

Chronic obstructive pulmonary disease (COPD) is a prevalent and debilitating disease characterised by persistent respiratory symptoms and limited airflow due to abnormalities in the airways or alveoli. This condition, primarily caused by significant exposure to noxious particles or gases, poses a considerable threat to global health. The Global Burden of Disease Study estimates COPD to be the third leading cause of death worldwide by 2020, emphasising the gravity of its morbidity and mortality rates [1]. The diagnostic criteria for COPD include a ratio of the forced expiratory volume in 1s (FEV1) to forced vital capacity (FVC) less than 0.7, confirming the presence of airflow limitation that is not fully reversible [2]. Global Initiative for Chronic Obstructive Lung Disease (GOLD) guidelines classify COPD into mild (FEV1 ≥ 80% predicted), moderate (50% ≤ FEV1 < 80%), severe (30% ≤ FEV1 < 50%), and very severe (FEV1 < 30%) disease [3].

The increasing prevalence of COPD has imposed a growing burden on surgeons and anaesthesiologists who now manage a larger volume of high-risk respiratory patients [4]. Especially when it comes to laparoscopic procedures, COPD patients present unique challenges. Traditional surgical approaches often leverage general anaesthesia (GA), where patients are rendered unconscious during surgery. However, emerging studies have suggested that regional anaesthesia (RA), a technique that numbs a specific part of the body, may be a superior alternative for certain patient populations [5].

Laparoscopic surgeries, which have surged in popularity over recent years, offer several advantages over traditional techniques, such as smaller incisions, reduced blood loss, less post-operative pain, and faster recovery. However, these procedures are not without their complications. In particular, the creation of a pneumoperitoneum, an artificial space in the abdominal cavity inflated with CO_2_, is fundamental for laparoscopic surgeries. This process, however, can lead to hypercapnia (an excess of CO_2_ in the bloodstream), increased intra-abdominal pressure (IAP), and decreased venous return, potentially precipitating cardiovascular instability [6,7].

Patients with advanced COPD are especially vulnerable when under GA with tracheal intubation and intermittent positive pressure ventilation (IPPV). The GA-associated increase in pulmonary vascular resistance, combined with the respiratory effects of pneumoperitoneum, can escalate the risk of pulmonary complications, including hypoxemia, laryngospasm, barotrauma, bronchospasm, and even cardiovascular instability [8]. Hence, there is a growing consensus that RA may serve as a safer choice for patients with COPD. Notably, spinal and epidural anaesthesia given at the lumbar level have been shown not to affect respiratory function, making them particularly attractive alternatives for this patient population [9]. However, the balance of benefits and risks between different anaesthetic techniques in COPD patients undergoing laparoscopic surgeries remains an area of ongoing research.

Despite a multitude of animal model studies on the physiological effects of pneumoperitoneum, the human body's specific response, particularly regarding changes in blood gases, respiratory, and venous systems, remains relatively unexplored. Moreover, the metabolic implications of pneumoperitoneum using CO_2_ are not well documented, especially in COPD patients from Western countries and India [10]. Therefore, our research aims to investigate the changes in heart rate, mean arterial pressure, arterial and end-tidal CO_2_, and bicarbonate (HCO_3_) during laparoscopic cholecystectomy in COPD patients, in order to contribute to the optimisation of anaesthetic management in this population.

## Materials and methods

Study design and setting

This was an observational, cross-sectional study conducted in a tertiary care hospital setting. The purpose was to investigate changes in heart rate (HR), mean arterial pressure (MAP), arterial and end-tidal CO_2_ (PaCO_2_ and EtCO_2_), and bicarbonate (HCO_3_) during laparoscopic cholecystectomy in patients with chronic obstructive pulmonary disease (COPD).

Participants

The participant pool consists of adult COPD patients (aged 35-65 years) who were diagnosed based on the Global Initiative for Chronic Obstructive Lung Disease (GOLD) criteria, with an FEV1/FVC ratio of less than 0.7 post-bronchodilator. GOLD 1 is classified as mild with FEV1 ≥ 80% predicted and GOLD 2 as moderate with 50% ≤ FEV1 < 80% predicted [3]. The participants were all scheduled for elective laparoscopic cholecystectomy. Patients with any contraindication for laparoscopic surgery, patients who convert to open surgery, those with cardiac diseases, or any other systemic disease were excluded from the study.

Anaesthesia technique

All the patients included in the study were educated about lung volume expansion manoeuvres like voluntary deep breathing and incentive spirometry preoperatively to institute it in the post-operative period for early recovery. A wide-bore cannula was taken, and intravenous fluid was started. Left or right radial artery cannulation with a 22-gauge cannula was performed under local anaesthesia after carrying out Allen’s test. Patients were then taken to the operating room, and standard monitoring was applied. Patients were preoxygenated using 100% oxygen for three to five minutes, and anaesthesia was induced with 0.05 mg/kg injection midazolam IV 1-2 μg/kg, injection fentanyl IV 2-3 mg/kg, injection propofol IV in suitable doses; following loss of verbal command, non-depolarising muscle relaxant injection vecuronium was given IV at a dose of 0.08-0.12 mg/kg bag and mask ventilation was done for three minutes followed by orotracheal intubation with suitable size endotracheal tube. Maintenance was done with 1%-2% isoflurane in a mixture of 50% oxygen/50% medical air with intermittent boluses of injection vecuronium and fentanyl. The pneumoperitoneum was created by peritoneal insufflation with carbon dioxide and maintains an abdominal pressure between 10 and 12 mm of Hg all the time. Laparoscopic cholecystectomy was performed according to a standard four-port technique in all the patients. Isoflurane was discontinued at the time of skin closure, and flows were increased to 10 litres/min with a fraction of inspired oxygen (FiO_2_) to 100%. One gram of injection paracetamol was given intravenously, and an injection of 0.25 bupivacaine was infiltrated at all four ports during closer. Residual muscle relaxation was antagonised with 0.01 mg/kg glycopyrrolate and 0.04-0.07 mg/kg neostigmine at the end of the operation.

Variables

The main variables studied include heart rate, mean arterial pressure, arterial and end-tidal CO_2_ (PaCO_2_ and EtCO_2_), and bicarbonate (HCO_3_). These are measured at baseline (before induction of anaesthesia), 30 minutes after induction or 15 minutes post-insufflation, 15 minutes post-deflation, and 60 minutes post-operative. Other variables, such as demographic data (age, sex, BMI), type of anaesthesia used, and perioperative variables (duration of surgery, intraoperative complications) are also collected for comprehensive analysis.

Data sources/measurement

Clinical assessments and measurements are made using standard hospital equipment and protocols. Arterial blood gases are collected and analysed using a blood gas analyser. End-tidal CO_2_ (EtCO_2_) is measured through capnography, a standard monitor used during anaesthesia.

Bias

All the patients undergoing laparoscopic cholecystectomy were evaluated on an objective checklist comprising the inclusion and exclusion criteria sets for this study. All patients fulfilling the criteria and consenting to participation were included in the study without fail to limit selection bias. Observer bias was limited by having a blinded assessor analyse the recorded data.

Study size

The study size was determined using power analysis based on previous studies examining similar physiological parameters during laparoscopic surgery in patients with COPD.

Statistical methods

Descriptive statistics are used to summarize the demographic and clinical characteristics of the patients. The correlation between HCO_3_ and EtCO_2_ was plotted on a scatterplot, and Pearson’s ‘r’ was calculated. The changes in physiological parameters over time are analysed using a paired t-test. A p-value of less than 0.05 is considered statistically significant. All analyses are conducted using a statistical software package.

Ethical considerations

The study protocol is approved by the hospital’s institutional ethics committee Dr. RMLIMS, Lucknow, with reference no. ICC No. 66/19, and all patients provided written informed consent before participation. The study is carried out in compliance with the principles of the Declaration of Helsinki and local regulations.

## Results

The study sample included 50 patients with COPD undergoing laparoscopic cholecystectomy. The mean age was 56 years, with a balanced gender distribution having 54% males and 46% females, and the mean body mass index (BMI) was approximately 28 kg/m^2^. Most patients (72%) had a GOLD stage II (moderate COPD), with the rest (28%) being stage I (mild COPD) (Table 1).

**Table 1:** Demographic variables BMI: body mass index, ASA: American Society of Anesthesiology, GOLD: Global Initiative for Chronic Obstructive Lung Disease.

S. No	Variables		Values	Values in Percentage (%)
1	Age in years (mean±SD) (minimum-maximum)		56.92±7.79 (35-65 yrs)	-
2	BMI in kg/m^2^ (mean±SD)		28.92±3.72	-
3	Gender (n=50)	Male	27	54%
		Female	23	46%
4	ASA physical status among patients (n=50)	ASA I	22	44%
		ASA II	28	56%
5	GOLD staging among patients (n=50)	GOLD I	14	28%
		GOLD II	36	72%

Throughout the procedure, heart rate (HR), mean arterial pressure (MAP), and end-tidal CO_2_ (EtCO_2_) showed a statistically significant increase from baseline at 15 minutes post-insufflation, i.e. 30 minutes post-induction (p-value < 0.05). However, these values returned to baseline levels within 15 minutes of deflation. During the study, a notable increase in HR was observed 30 minutes after induction of anaesthesia, i.e. from 81.21±6.17 to 93.82±6.92 with a mean difference of -12.60 (p-value < 0.001), which normalised 60 minutes post-operatively (p-value > 0.05). MAP showed little variation over time except at 15 minutes post-insufflation when it was significantly increased (p-value < 0.05) (Table 2).

**Table 2:** Showing changes in heart rate (HR) and mean arterial pressure (MAP) over time

S. No	Time (in minutes)	Changes in Heart Rate (HR) Over Time (in beats per minute)			Changes in Mean Arterial Pressure (MAP) Over Time (in mm of Hg)		
		Mean±SD	Mean differences	p-value (p < 0.05 is significant)	Mean±SD	Mean differences	p-value (p < 0.05 is significant)
1	Pre-operative (baseline)	81.21±6.17	-	-	78.47±8.97	-	-
2	30 min after induction or 15 min post-insufflation	93.82±6.92	-12.60	<0.001	83.89±6.54	-5.42	<0.05
3	15 min post-deflation	83.72±7.09	-2.51	>0.05	80.57±7.18	-2.10	>0.05
4	Post-operative after 60 min	80.89±5.63	0.32	>0.05	79.76±6.08	-1.29	>0.05

Blood pH showed a small but statistically significant decrease at 30 minutes after induction, i.e. 15 minutes post-insufflation from 7.35±0.17 to 7.29±0.13 with a mean difference of 0.068 (p-value < 0.001) but recovered at somewhat 60 minutes post-operatively, while bicarbonate (HCO_3_) levels showed slight increase all over time from baseline but not significant (p-value>0.05) always within the normal physiological range (Table 3).

**Table 3:** Showing changes in pH and HCO3 over time pH: potential of hydrogen, HCO_3_: bicarbonate.

S. No	Time (in minutes)	Changes in pH Over Time			Changes in HCO_3_ over time (in mmol/L)		
		Mean ± SD	Mean differences	p-value (p < 0.05 is significant)	Mean ± SD	Mean differences	p-value (p < 0.05 is significant)
1	Pre-operative	7.35±0.17	-	-	20.78±2.17	-	-
2	30 min after induction or 15 min post-insufflation	7.29±0.13	0.068	<0.001	21.22±1.44	-0.44	>0.05
3	15 min post-deflation	7.31±0.13	0.04	>0.05	21.34±1.75	-0.56	>0.05
4	Post-operative after 60 min	7.32±0.08	0.03	>0.05	21.48±2.07	-0.70	>0.05

The end-tidal CO_2_ (EtCO_2_) showed a statistically significant increase from baseline at 15 minutes post-insufflation or 30 minutes after induction, i.e. from 36.50±4.29 to 39.51±3.39 with a mean difference of -3.01 (p-value < 0.001). However, these values returned to baseline levels within 15 minutes of deflation and decreased to near pre-operative levels 60 minutes post-operatively. Arterial CO_2_ (PaCO_2_) levels showed fluctuation over the course of the procedure, at 15 minutes post-insufflation, i.e. 30 minutes after induction, it increased significantly from baseline 43.45±4.11 to 45.56±3.67 with a mean difference of -2.11 (p-value < 0.05) which became non-significant after 15 minutes of deflation and 60 minutes post-operatively (Table 4).

**Table 4:** Showing changes in PaCO2 and EtCO2 over time PaCO_2_: partial pressure of arterial carbon dioxide, EtCO_2_: end-tidal carbon dioxide.

S. No	Time (in minutes)	Changes in PaCO_2_ Over Time (in mm of Hg)			Changes in EtCO_2_ Over Time (in mm of Hg)		
		Mean ± SD	Mean differences	p-value (p < 0.05 is significant)	Mean ± SD	Mean differences	p-value (p < 0.05 is significant)
1	Pre-operative	43.45±4.11	-	-	36.50±4.29	-	-
2	30 min after induction or 15 min post-insufflation	45.56±3.67	-2.11	<0.05	39.51±3.39	-3.01	<0.001
3	15 min post-deflation	44.14±4.27	-0.69	>0.05	37.62±4.13	-1.12	>0.05
4	Post-operative after 60 min	43.84±3.32	-0.39	>0.05	35.96±4.28	0.54	>0.05

**Figure 1:**
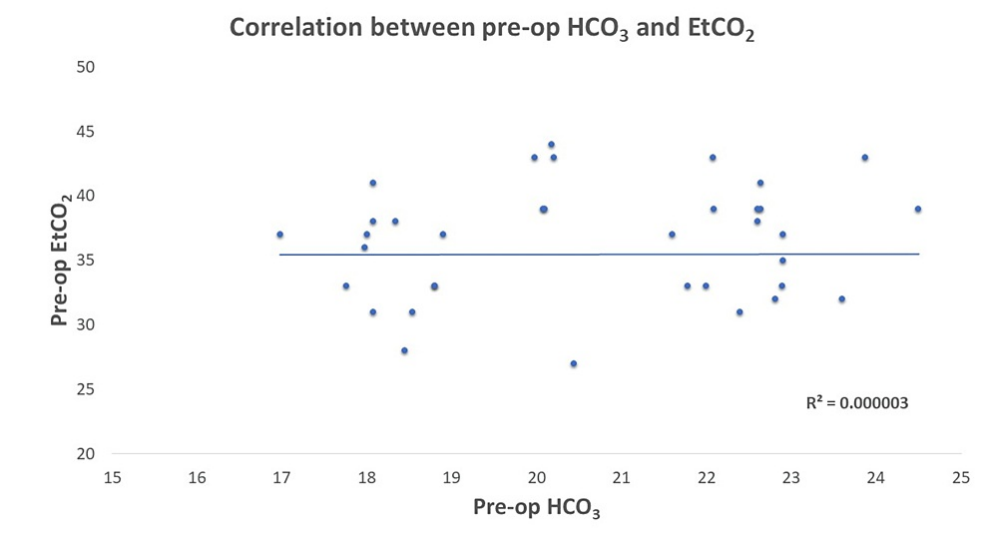
Shows no correlation of pre-operative EtCO2 and HCO3 Pre-op: pre-operative, EtCO_2_: end-tidal carbon dioxide, HCO_3_: bicarbonate.

**Figure 2:**
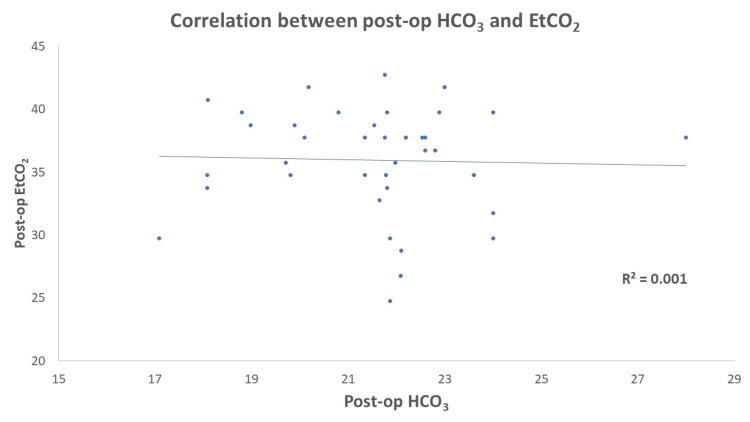
Shows no correlation of post-operative EtCO2 and HCO3 Post-op: post-operative, EtCO_2_: end-tidal carbon dioxide, HCO_3_: bicarbonate.

EtCO_2_ and PaCO_2_ levels showed a significant increase 15 minutes post-insufflation (p-value<0.05), but these changes were clinically insignificant as they remained within the normal physiological range. There was no significant correlation between pre- and post-operative EtCO_2_ and HCO_3_ with a Pearson correlation ‘r’ of 0.002 and 0.032 (Table 5, Figures 1, 2).

**Table 5:** Correlation of pre- and post-operative EtCo2 and HCO3 EtCO_2_: end-tidal carbon dioxide, HCO_3_: bicarbonate.

S. No	Time Interval	EtCO_2_ (in mm of Hg) Mean±SD	HCO_3_ (in mmol/L) Mean±SD	Pearson Correlation ‘r’
1	Pre-operative	31.50±4.29	20.78±2.17	0.002
2	Post-operative	32.21±4.28	21.48±2.07	0.032

Complications were minimal, with only one case of delayed emergence and one case of bronchospasm among the studied patients. No serious perioperative complications were observed, and all patients had an uneventful recovery.

## Discussion

The results of this study confirm our hypothesis that laparoscopic cholecystectomy could lead to transient physiological changes in COPD patients, especially during the period of insufflation. The observed changes in heart rate, mean arterial pressure, and end-tidal CO_2_ are consistent with previous studies [11]. Wittgen et al. studied that patients with pre-operative cardiopulmonary disease showed a significant increase in arterial blood pressure (p-value < 0.05) and a decrease in pH during CO_2_ pneumoperitoneum compared with patients without underlying disease [11]. Joris et al. demonstrated during laparoscopic surgery that both mechanical and humoral factors contribute to an increase in systemic vascular resistance [12]. Similarly, Muralidhar postulated increased systemic vascular resistance, increased mean arterial pressure, and minimal increase in heart rate during pneumoperitoneum [13].

The slight elevation in arterial CO_2_ and bicarbonate levels suggests a transient hypercapnic response due to CO_2_ absorption from the pneumoperitoneum and the slightly compromised ability of COPD patients to eliminate CO_2_ due to impaired venous return and increased dead space ventilation caused by pneumoperitoneum as Liu et al. demonstrated an increase in PaCO_2_ at the end of the procedure from 4.43 to 5.81 kPa despite an unstated increase in ventilation [14]. Wittgen et al. noted that patients with cardiopulmonary disease might develop respiratory acidosis despite aggressive hyperventilation [11].

In their comparative study, Ozyuvaci et al. reported that PaCO_2_ increased during insufflation and reached a maximum at extubation (p-value < 0.05) [15]. It declined within 20 minutes post-operatively but did not return to pre-operative levels during this time in patients with COPD undergoing laparoscopic cholecystectomy. Bayrak and Altıntas reported that the PaCO_2_ at the post-operative five and 20 minutes were higher but not significant (p-value < 0.05) in the COPD patients undergone laparoscopic cholecystectomy under general anaesthesia [16].

Hakeem et al. and Iqbal et al. also reported significant changes in PaCO_2_, EtCO_2_, pH, and bicarbonate at 15-minute and 30-minute intervals (p-value < 0.05) with the return to baseline in the early post-operative period [8,17]. There was a slight recovery of post-operative pH (7.32±0.08) from the intra-operative phase (7.29±0.13) and approaching towards the pre-operative value (7.35±0.17), suggesting that COPD patients are able to maintain their acid-base balance even during the stress of surgery and pneumoperitoneum.

We observed no correlation between pre- and post-operative EtCO_2_ and HCO_3_. Baraka et al. reported that the EtCO_2_ increased with time after induction and insufflation to reach a maximum value after 40 minutes and correlated with the baseline value prior to carbon dioxide insufflation in a positive linear relationship [18]. However, these changes were not clinically significant, which aligns with the existing research suggesting that laparoscopic surgery is safe in patients with COPD.

It should also be highlighted that only a few complications, such as delayed emergence and bronchospasm in one case each, were observed in our study population. This low incidence of complications could be attributed to meticulous perioperative care and strict adherence to anaesthetic protocol. Our study's strength lies in its prospective design and the robustness of the measurement of variables.

Limitation

It is an open-labelled observational study, so complete exclusion of bias cannot be ensured. It is also important to note that our results may not generalise to patients with severe COPD, as our sample predominantly consisted of patients with mild to moderate COPD.

## Conclusions

Finally, while our study sheds light on the physiological changes during laparoscopic cholecystectomy in COPD patients, it also underscores the need for further research on strategies to minimize these changes and enhance the safety of this patient population during laparoscopic surgeries.
